# Molecular cytogenetic characterization of canine histiocytic sarcoma: A spontaneous model for human histiocytic cancer identifies deletion of tumor suppressor genes and highlights influence of genetic background on tumor behavior

**DOI:** 10.1186/1471-2407-11-201

**Published:** 2011-05-26

**Authors:** Benoit Hedan, Rachael Thomas, Alison Motsinger-Reif, Jerome Abadie, Catherine Andre, John Cullen, Matthew Breen

**Affiliations:** 1 Department of Molecular Biomedical Sciences, College of Veterinary Medicine, North Carolina State University, Raleigh, NC, USA; 2 Center for Comparative Medicine and Translational Research, North Carolina State University, Raleigh, NC, USA; 3 Bioinformatics Research Center, North Carolina State University, Raleigh, NC, USA; 4 Department of Statistics, North Carolina State University, Raleigh, NC, USA; 5 UMR 707 IECM, AMaROC team, ONIRIS, Nantes, FRANCE; 6 Institut de Génétique et Développement, UMR 6061 CNRS/Université de Rennes1, Faculté de Médecine, Rennes, FRANCE; 7 Department of Population Health and Pathobiology, College of Veterinary Medicine, North Carolina State University, Raleigh, NC, USA; 8 Cancer Genetics Program, UNC Lineberger Comprehensive Cancer Center, Chapel Hill, NC 27599, USA

## Abstract

**Background:**

Histiocytic malignancies in both humans and dogs are rare and poorly understood. While canine histiocytic sarcoma (HS) is uncommon in the general domestic dog population, there is a strikingly high incidence in a subset of breeds, suggesting heritable predisposition. Molecular cytogenetic profiling of canine HS in these breeds would serve to reveal recurrent DNA copy number aberrations (CNAs) that are breed and/or tumor associated, as well as defining those shared with human HS. This process would identify evolutionarily conserved cytogenetic changes to highlight regions of particular importance to HS biology.

**Methods:**

Using genome wide array comparative genomic hybridization we assessed CNAs in 104 spontaneously occurring HS from two breeds of dog exhibiting a particularly elevated incidence of this tumor, the Bernese Mountain Dog and Flat-Coated Retriever. Recurrent CNAs were evaluated further by multicolor fluorescence *in situ *hybridization and loss of heterozygosity analyses. Statistical analyses were performed to identify CNAs associated with tumor location and breed.

**Results:**

Almost all recurrent CNAs identified in this study were shared between the two breeds, suggesting that they are associated more with the cancer phenotype than with breed. A subset of recurrent genomic imbalances suggested involvement of known cancer associated genes in HS pathogenesis, including deletions of the tumor suppressor genes *CDKN2A/B*, *RB1 *and *PTEN*. A small number of aberrations were unique to each breed, implying that they may contribute to the major differences in tumor location evident in these two breeds. The most highly recurrent canine CNAs revealed in this study are evolutionarily conserved with those reported in human histiocytic proliferations, suggesting that human and dog HS share a conserved pathogenesis.

**Conclusions:**

The breed associated clinical features and DNA copy number aberrations exhibited by canine HS offer a valuable model for the human counterpart, providing additional evidence towards elucidation of the pathophysiological and genetic mechanisms associated with histiocytic malignancies. Extrapolation of data derived from canine histiocytic disorders to human histiocytic proliferation may help to further our understanding of the propagation and cancerization of histiocytic cells, contributing to development of new and effective therapeutic modalities for both species.

## Background

Histiocytic malignancies in human patients are rare but aggressive cancers associated with high mortality [1-4]. Pathologic and cytogenetic data for these malignancies are sparse, based on a few early case studies e.g.[5-7] and a single larger study of 18 histiocytic sarcomas (HS) [2]. On a molecular level, deletions of *CDKN2A/p14ARF, TP53, MDM2 *and *PTEN *have been reported in human histiocytic disorders [4,8-14], but their etiology remains poorly understood. The clinical behavior of these diseases is also unclear, and the optimal course of treatment remains a matter of debate [4,15]. Elucidation of the genetic basis of many human cancers has been aided by identification of recurrent genomic DNA copy number aberrations (CNAs) affecting dosage of target genes involved in cancer pathogenesis. The diagnostic, prognostic and therapeutic significance of numerous CNAs in a variety of common cancers is well described [16]. However, for rare cancers, including HS, the limitations on sample availability preclude the generation of comprehensive data regarding recurrent CNAs.

Histiocytic cancers are uncommon within the domestic dog population in general, but there is a highly elevated incidence in several breeds, including the Bernese Mountain Dog (BMD) and Flat-Coated Retriever (FCR), suggesting heritable risk factors and indicating that these breeds may share genetic characteristics contributing to tumor initiation and progression [17-22]. Canine HS are histologically comparable to the corresponding human cancers, involving proliferation of members of both histiocytic lineages (dendritic cells (DC) and macrophages) with which they share pathologic features [17,18]. With only small numbers of available human samples, we propose that the canine model provides a unique opportunity to identify recurrent genomic lesions associated with spontaneous HS, and provide greater insight into the pathogenesis and genetic etiology in human patients.

For this study we hypothesized that recurrent CNAs exist in canine HS, detection of which would identify regions of the canine genome containing genes associated with HS initiation and progression. Approximately ~25% of all tumors diagnosed in the BMD are reported to be HS, and a recent study estimated that 80% of canine disseminated HS cases are diagnosed in the BMD, suggestive of a multigenic or multifactorial mode of transmission [22,23]. The typical age of onset in the BMD is 6.5 years, with 82% and 55% of cases involving an internal organ and multiple organs, respectively [22,23]. This latter presentation represents the disseminated form of the disease, often referred to as malignant histiocytosis. Tumor progression is rapid with a mean survival time following diagnosis of only 49 days [23]. With such an aggressive behavior and high prevalence in the breed, HS has a huge impact on BMD longevity. HS also is the most common malignant tumor identified in the FCR, accounting for at least 40% of all tumors diagnosed in this breed, with an average age of onset of 8.5 years [24,25]. Tumors in the FCR are generally located in the muscle region surrounding a joint, with a high rate of metastasis to local lymph nodes, spleen, thorax and abdominal organs. While treatments are available for palliation of clinical signs and extension of life, this tumor carries a poor prognosis in the FCR, with a reported median survival of only four months [25].

We evaluated genome-wide CNAs in a cohort of histologically confirmed canine HS cases using array comparative genomic hybridization (aCGH), supplemented with fluorescence *in situ *hybridization (FISH) and loss of heterozygosity (LOH) analysis. We identified recurrent CNAs common to HS in both breeds (BMD and FCR), indicating an association with tumor phenotype. These changes included genomic imbalances encompassing well defined cancer associated genes (*CDKN2A/B, RB1, PTEN*). Epidemiological data revealed a significant difference in the anatomical location of histiocytic tumors between the two breeds. A subset of CNAs was also associated significantly with breed. These data suggest that at least some of the CNAs are associated with breed and/or tumor location, rather than tumor phenotype.

Having defined aberrant genomic regions in two breeds of dog with a high incidence of HS, subsequent comparative molecular analysis of such regions, both in dog and human patients, will provide opportunities to gain greater insight into our understanding of the pathways implicated in histiocytic cancers, providing a first step on the road to developing new treatments.

## Methods

### Case recruitment and histological evaluation of canine histiocytic tumors

No animal experimentation was performed during this study. All patients evaluated in this study were from family owned dogs with a confirmed histiocytic malignancy. All blood and tumor samples were taken with informed owner consent by veterinarians between 2003-2008. One hundred and forty six patients were recruited for this study. Unfixed tumor biopsies were submitted from 125 cases that had not previously received treatment for their HS other than for palliative care. Tumor biopsies were obtained under sterile conditions, either as part of a routine diagnostic biopsy procedure, during surgery, or immediately following euthanasia. All FCR cases (n = 45) originated from the USA, while the BMD cases (n = 101) were derived from the USA (n = 68 patients) or France (n = 33 patients). The anatomical location of tumors evident at the time of diagnosis and/or necropsy was recorded for each case in one of five categories: tumor present in i) one internal organ; ii) multiple internal organs (equivalent to disseminated HS); iii) lymph node only; iv) limb only; v) skin only. The last two locations were regarded as localized HS. A representative portion of the tumor was fixed in 10% neutral-buffered formalin. Histological specimens were evaluated by board-certified veterinary pathologists (JC, JA) using routine hematoxylin-eosin (H&E) staining and antibodies against CD3 (T-cell marker), CD18 (hematopoietic marker), CD79a (B-cell marker), MHC class II, E-cadherin and Thy-1. In rare cases where the pathology remained inconclusive (and where frozen tissue was available) tissues were evaluated further with CD11c and CD11d. In such cases, a diagnosis of HS was confirmed when tumor cells were positive for CD11c or CD11d markers and negative for CD3 and CD79a markers. Tumors were classified according to the criteria of Affolter and Moore (2002)[17].

### Array comparative genomic hybridization (aCGH) analysis

Genomic DNA was isolated from representative specimens of unfixed tumor tissue. aCGH analysis was performed as described previously [26] using a custom genomic microarray comprising canine bacterial artificial chromosome (BAC) clones distributed at ~1 Mb intervals within the 7.6× canine genome sequence assembly [27]. All BAC clones had been previously verified to map to a unique chromosomal location by multicolor fluorescence *in situ *hybridization (FISH) analysis [26]. Equimolar quantities of blood-derived DNA isolated from five or more unrelated, cancer free individuals were used as breed matched reference samples. Data analysis was performed as described elsewhere [26,28]. Tumor-associated genomic imbalances were detected using the aCGH-Smooth algorithm [29] with threshold limits for detection of CNAs set at log_2 _ratio values of tumor DNA vs. reference DNA equivalent to 1.15:1 (copy number gain) and 0.85:1 (copy number loss). CNAs were defined as recurrent or highly recurrent when common to ≥30% and ≥50% of cases, respectively. The megabase (Mb) location of dog genes along the corresponding chromosome were based on the canFam v2 genome sequence assembly [27] accessed via the UCSC genome browser http://genome.ucsc.edu/.

### Loss of heterozygosity (LOH) analysis

Genomic regions exhibiting recurrent DNA copy number loss in HS were further evaluated for LOH using the M13-tailed primer method [30]. PCR primers flanking microsatellite sequences [31] located within these regions are listed in Additional file 1: Table S1. Each locus was amplified independently from paired tumor- and blood-derived DNA from each patient evaluated. PCR was conducted in 10 μl reactions containing 0.8 units Taq polymerase (Go Taq, Promega), 1.0 μl 10× reaction buffer (Promega), 0.25 mM each dNTP, 0.15 μM each microsatellite-specific primer, 0.1 μM 5' fluorescently labeled M13 primer and 50 ng template DNA. The M13 primer was tagged at the 5' end either with PET, VIC, FAM or NED (Applied Biosystems) to facilitate multiplexing of products. Amplification conditions were as follows: 95°C for 2 min followed by 35 cycles of 94°C for 30 s, 58°C for 30 s and 72°C for 30 s, followed by a 2-min final extension at 72°C. Amplicons were visualized and evaluated by capillary-electrophoresis (3730xl DNA Analyzer, Applied BioSystems). Two parameters were calculated for each sample: the allelic ratio (AR) and allelic balance (AI). The AR was calculated by AR = (peak area 1)/(peak area 2); AI by AI = AR (tumor)/AR (blood). When AI ≥ 1.5 or ≤ 0.67, the region was considered to be deleted.

### Cytogenetic and fluorescence *in situ *hybridization (FISH) analysis

Where viable tumor tissue was available, interphase nuclei and chromosome preparations were generated for FISH analysis. Primary tumor specimens were disaggregated using Collagenase B (Roche) and the resulting cell suspensions harvested directly, or from low passage (n <2) primary cell cultures, using conventional techniques of colcemid arrest, hypotonic treatment and methanol/glacial acetic acid fixation, as described elsewhere [32]. Multicolor FISH analysis was carried out as described previously [33] using BAC clones representing regions of the genome highlighted by aCGH analysis. All probes were hybridized first onto metaphase chromosome preparations from a pool of clinically healthy dogs to confirm the expected copy number for each probe at the expected chromosomal location. Image data were assessed from a minimum of 30 representative cells from each control/case evaluated.

### Statistical analysis

aCGH, clinical and demographic data were compared with Mann-Whitney U tests in the case of continuous outcome variables, and with Fisher's Exact tests in the case of categorical outcomes. These methods are nonparametric, requiring no distributional assumptions to retain validity. Principal components analysis (PCA) is well-established in human genetics to detect geographic and racial background differences in human populations [34,35]. PCA was performed to evaluate potential population substructure in the USA and French BMD populations, and to test for differences in CNA frequency between the two breeds [36-38]. Association analyses were performed with Fisher's Exact tests to test for association between aberration frequencies and breed. To control family-wise error rates and correct for multiple comparisons, permutation testing was performed, deriving empirically p-value cut-offs of significance corresponding to a family-wise error rate of 0.05 [39]. Statistical analyses were performed using JMP Genomics v4 and SAS 9.1.3 (SAS Institute, Cary, NC) and Stata v.11 http://www.stata.com.

## Results

### Statistical evaluation of epidemiological data

**Figure 1:**
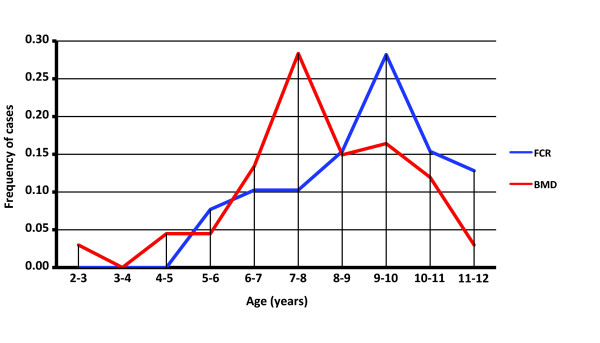
**Distribution of the age of diagnosis of HS in US-resident BMD (68 cases) and FCR (45)**. The mean age of HS diagnosis is significantly different between BMD (7.7 yrs) and FCR (8.6 yrs) (pval = 0.01, Mann-Whitney U test).

Evaluation of the 113 HS cases recruited from within the USA provided an opportunity for direct comparison of their epidemiological characteristics. The USA cohort comprised 68 BMD (33 male, 35 female) and 45 FCR (20 male, 25 female), all of whom were registered with the American Kennel Club. The mean age at diagnosis was 8.6 years ± 1.7 for the FCR (range 5 to 12 years) and 7.7 years ± 1.9 for the BMD (range 2 to 12 years), which also showed a secondary peak at 10 years of age (Figure 1). These data indicate that within the US patients the age of onset of HS was significantly higher in the FCR than the BMD (p = 0.01, Mann-Whitney U test).

**Figure 2:**
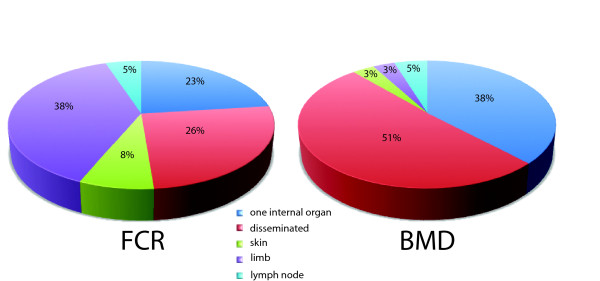
**Anatomical distribution of histiocytic tumors in FCR and BMD**. Anatomical location of HS is significantly different between the two breeds (p value < 0.001, Fisher Exact test).

The anatomical location of the tumor(s) also showed significant variation between the two breeds (Figure 2), with 87% of BMDs presenting with HS affecting one or more internal organs compared with 48% of FCRs (p < 0.001, Fisher's Exact test). Moreover, occurrence of HS on a limb was >10 times more frequent in the FCR than in the BMD (38.4% versus 3.2%). With the assumption that isolated skin and limb tumors correspond to localized HS, we may surmise that the prevalence of localized HS is seven times more frequent in the FCR than in the BMD (46.1% versus 6.5%) and that of disseminated HS is approximately two fold higher in the BMD than the FCR (50.8% versus 25.6%).

### Overview of DNA copy number aberrations revealed by aCGH

Metaphase preparations were generated from >20 HS tumor biopsies from both BMD and FCR. The domestic dog karyotype comprises 38 pairs of single-armed chromosomes and a pair of bi-armed sex chromosomes (2n = 78). Conventional cytogenetic evaluation of HS cases revealed highly variable chromosome numbers in both breeds, which were generally in the range 42-58. All cases evaluated exhibited an abundance of aberrant bi-armed chromosomes. These data suggest that in addition to numerical changes there also are large numbers of structural changes that merit further evaluation in a subsequent study.

Of the 146 HS cases available to this study (68 BMD and 45 FCR from the USA, and 33 BMD from France), unfixed tumor biopsies were obtained for 125 (Additional file 2: Table S2). Tumor specimens from 104 cases (33 FCRs, 71 BMDs) yielded DNA of sufficient quality to permit aCGH analysis, of which 86 (82.6%) (30 FCRs, 56 BMDs) presented with detectable CNAs. The remaining 18 cases (17.3%) (3 FCR and 15 BMD) did not demonstrate any detectable CNAs at 1 Mb resolution, presumably either due to an abundance of non-malignant tissue in the biopsy received, or the presence of a highly polyclonal cell population with few shared aberrations. Since these 18 cases provided no evidence for CNAs they were excluded from subsequent analyses.

**Figure 3:**
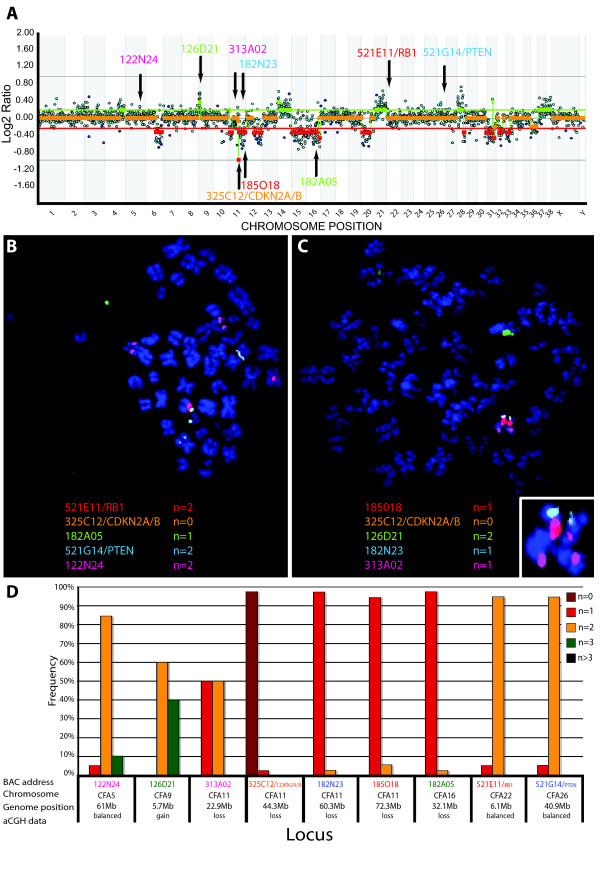
**Molecular cytogenetic evaluation of a canine histiocytic malignancy using aCGH and FISH.** A. Example of whole genome aCGH profile of a HS in a five year old female FCR. Log_2 _ratios representing thresholds of genomic gain and loss are indicated by horizontal bars above (green line) and below (red line) the midline (orange line), which represents normal copy number. The chromosome copy number status line for the tumor appears as an orange overlay of the center-line when there is a normal copy number, and as either green (gain) or red (loss) in the regions where genomic imbalances were apparent, as determined by the aCGH Smooth algorithm [29]. The aCGH profile is annotated with the clone address of nine BAC clones from the 1 Mb array that were used in subsequent FISH analysis of this case. Three of these nine clones have been shown previously to contain the full coding sequence of a key cancer-associated gene (*CDKN2A*, *RB1*, *PTEN*) [26]. The color of the text denotes the fluorochrome with which the BAC clone was labeled. **B, C **Targeted FISH analysis of tumor metaphase chromosome spreads from the same case using nine differentially labeled BAC clones (highlighted in **A**) combined in two separate groups. The modal copy number for each clone is indicated. **D**. Summary of copy number data of all nine loci evaluated by FISH analysis of at least 30 tumor interphase nuclei or metaphase spreads. The aCGH copy number status of these regions (gain, loss, balance) are indicated, demonstrating concordance between FISH data and aCGH data.

**Figure 4:**
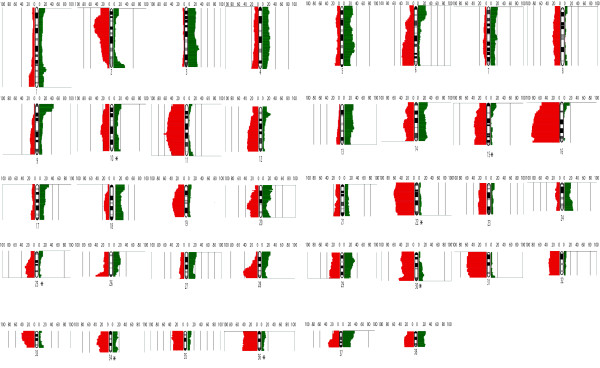
**Whole genome CNA penetrance plot showing the percentage of all HS cases in the cohort (BMD+FCR; n = 86 cases) that presented with detectable DNA copy number gain (green) and loss (red) along the length of each dog chromosome (CFA 1 to CFA 38), surveyed at 1 Mb intervals**. The asterisks indicate those seven chromosomes (CFA 10, 15, 22, 25, 30, 35 and 36) that have regions of CNAs differing significantly between BMD and FCR.

**Figure 5:**
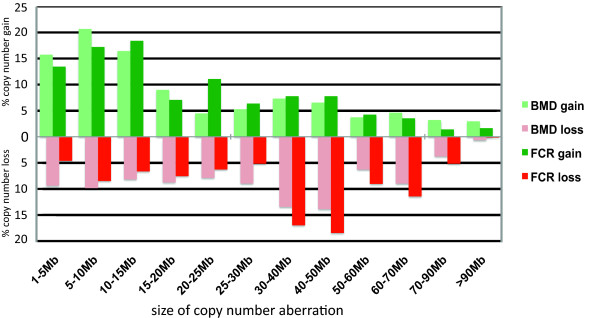
**Distribution of the size of the CNAs identified by aCGH analysis of HS of BMD and FCR**.

Typically, aCGH profiles for individual canine HS cases demonstrated numerous CNAs, both gains and losses, throughout the genome (Figure 3a), which were supported by FISH analysis of select regions (Figures 3b, c and 3d). When considered as a single population of 86 aberrant cases, the genome wide aCGH profiles for canine HS shared numerous CNAs (Figure 4). Thirty-one regions of the canine genome presented with recurrent DNA copy number increases (present in ≥30% of the combined cohort), comprising eight regions of gain and 23 regions of loss. Of these 31 regions, six were highly recurrent (present in ≥50% of the combined cohort), all of which were deletions; located on dog chromosome 2 (*Canis familiaris*, CFA 2) (50% cases), CFA 11 (62.8% of cases), CFA 16 (86% of cases), CFA 22 (64% of cases) and CFA 31 (61.6% of cases). Overall the mean number and size of CNAs in both breeds was highly comparable (Table 1), although the proportion of cases that showed no detectable CNAs was 2.3 fold greater in the BMD (21%) than in the FCR (9%). Figure 5 shows the size distribution of regions of CNA within the FCR and BMD, indicating that in both breeds more than 50% of the observed deletions were >30 Mb in size, while half of the gains were <15 Mb.

**Table 1:** Summary of the overall DNA copy number status in the study population.

	BMD	FCR	Mean (+/- st.dev)
mean number of CNAs	30.3 ± 17.8	32.2 ± 17.8	30.7 ± 17.6
mean number of losses	15.9 ± 9.3	18.1 ± 7.2	16.7 ± 8.6
mean number of gains	13.8 ± 10.3	14.1 ± 12.3	13.9 ± 10.9
ratio losses:gains	1.15:1	1.27:1	1.22:1
mean size of loss (Mb)	30.7 ± 21	35.7 ± 20.5	32.5 ± 21
mean size of gain (Mb)	23.3 ± 23.7	22.4 ± 20.5	23.1 ± 22.6

(CNAs = Copy Number Aberrations, BMD = Bernese Mountain Dog, FCR = Flat Coated Retriever, Mb = Megabase).

### Identification of recurrent population associated aberrations

**Figure 6:**
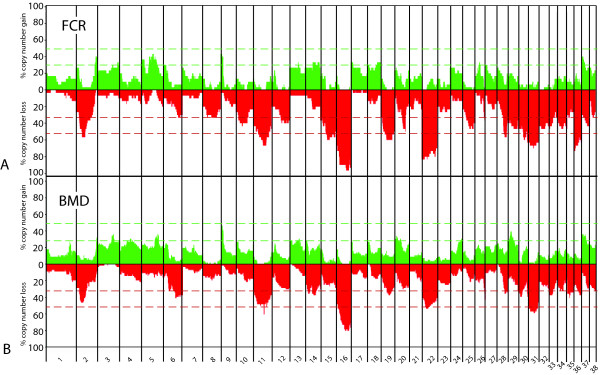
**Whole genome CNA penetrance plots separated by breed A) FCR and B) BMD, where each indicates percentage of HS cases identified with DNA copy number gain (green) and deletion (red) along the length of each dog chromosome (CFA 1 to CFA 38) in 1 Mb intervals**. The dashed horizontal red and green lines indicate thresholds of recurrence (≥33% incidence) and high recurrence (≥50% incidence).

**Figure 7:**
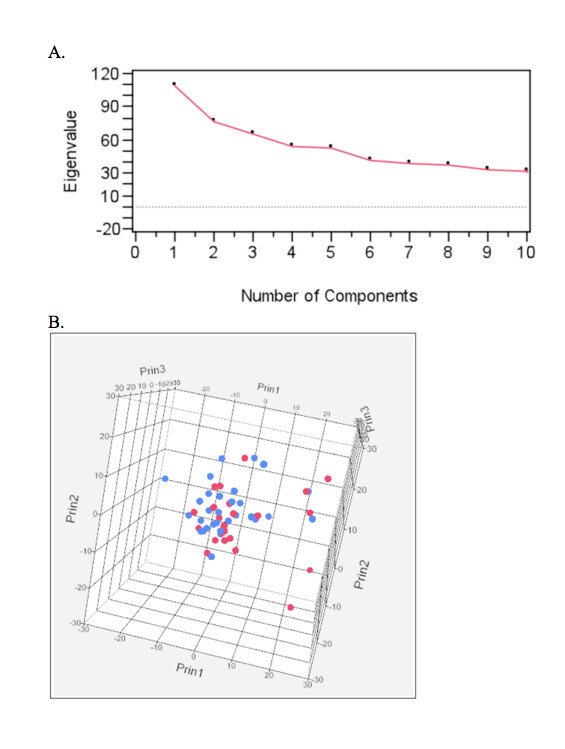
**Principal Component Analysis (PCA) to identify potential population substructure between French and American BMD cases of HS**. A). Scree plots of the eigen values first 10 components. B). Three dimensional plot of the first three components. American BMD patients are indicated in red while French BMD patients are indicated in blue.

Segregation of aCGH data by breed revealed remarkable genome-wide similarity in the gross distribution of genomic gains and losses between BMD and FCR (Figure 6), indicating that most of the CNAs identified were common to both breeds. To identify whether the geographical origin of a patient (USA or France) had any significant effect on its genome wide CNA profile, principal component analysis (PCA) was used to investigate potential population substructure within the BMD cohort. Visual inspection of the scree plot of the first ten potential components indicated just a single significant component (Figure 7a). Eigen values for the first three components are represented in three-dimensional space in Figure 7b, which demonstrates that there is no division between BMD patients by geographical origin. Mann-Whitney U tests showed no significant association between the eigen values from the first five components and the geographic origin of the patient (p > 0.05 for each test). These results indicate there is no substantial population substructure between American and French BMDs. Based on these results the American and French BMDs were evaluated as one single population in subsequent association analysis.

**Figure 8:**
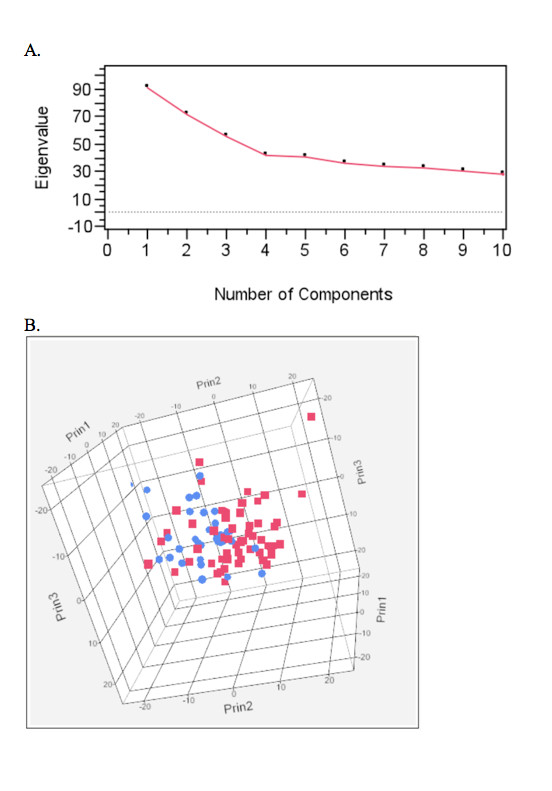
**Principal Component Analysis (PCA) to assess global variation in aberration frequency between BMD and FCR**. A) Scree plots of the eigenvalues first 10 components. B) Three dimensional plot of the first three components. BMD patients are indicated in red and FCR patients are indicated in blue.

PCA was also used to assess evidence for global differences in the distribution of genome-wide DNA copy number aberrations between the two breeds (Figure 8a). These results indicate four significant components that define the global data. The eigen values were generated corresponding to the first four components and were tested for association with breed using nonparametric Mann-Whitney U tests (Table 2). The results of these association tests indicate a statistically significant association between breed and the second principal component (p < 0.0001). Figure 8b shows that there is a strong division between the two breeds defined by the second component, visually representing the association demonstrated in Table 2. Tumor location was also tested for association with the eigen values for the first four principal components (Table 2), the results indicating a significant association between tumor location and the second principal component. Since there is a highly significant association between breed and tumor location, it is not possible to determine if it is the breed or the tumor location that is driving the association and so this must be considered when interpreting the results of breed association with CNA.

**Table 2:** Test of association between the first eigenvalues for the first four principal components and breed (BMD/FCR) or tumor location (internal/external).

Component	P-value for association test with breed	P-value for association test with tumor location
1	0.4174	0.08205431
2	<0.00001	7.12E-05
3	0.1291	0.06926817
4	0.2995	0.82846813

Fisher's exact tests of associations were performed for each region of DNA copy number gain or loss and breed, to identify specific regions of aberrations that define this global difference (Table 3). The permutation distribution indicated that an uncorrected p-value < 0.01 was statistically significant at a family-wise error rate (FWER) of 0.05. Copy number aberrations involving 13 regions on seven different chromosomes (CFA 10, 15, 22, 25, 30, 34, and 36) were significantly associated with breed.

**Table 3:** Thirteen regions along seven dog chromosomes showed significant differences between BMD and FCR tumors.

CFA	position (Mb)	P-value
10	25-30	<0.0057
15	19-22	<0.0063
15	29-49	<0.0094
15	52-54	<0.0059
22	00-12	<0.0023
22	31-48	<0.0089
22	54-57	<0.0064
25	09-12	<0.0046
25	14-18	<0.0062
30	26-28	<0.0040
30	30-33	<0.0076
34	45-46	<0.0094
36	00-20	<0.0099

The megabase (Mb) position of these regions on different chromosomes (CFA) is indicated. The uncorrected p-value is shown.

### Evaluation of tumor suppressor gene deletions in canine HS

A subset of recurrent CNAs identified in this study involved regions of the genome that contain known cancer associated genes and were investigated further. Deletion of CFA 11q16 at ~44 Mb, which includes the tumor suppressor gene *CDKN2A/B*, was identified in 62.8% of HS cases (60.7% of BMD and 66.7% of FCR) (Figure 6). This region was further evaluated by LOH analysis of 26 BMD and 20 FCR cases, each of which exhibited CFA 11q16 deletion in aCGH analysis, and had high quality DNA available from both peripheral blood and tumor specimens. Genotyping of seven microsatellites surrounding the *CDKN2A/B *locus demonstrated LOH of at least one microsatellite within this region in all 46 cases, consistent with the loss of CFA 11q16 identified by aCGH (data not shown). Moreover, LOH analysis of an additional 11 cases, in which loss of this region of CFA 11 was not apparent from aCGH analysis, revealed that six (54%) showed LOH of at least one marker close to the *CDKN2A/B *locus (data not shown). The common region deleted in both breeds was centered on the *CDKN2A/B *locus. Two of the cases used for this analysis showed log_2 _tumor DNA:reference DNA ratios < -1.0 at the *CDKN2A/B *locus, highly suggestive of an homozygous deletion. Subsequent FISH analysis using a BAC clone containing the *CDKN2A/B *locus confirmed homozygous deletion of this region in these tumors (see Figure 3 for an example).

The most frequent DNA copy number aberration observed by aCGH in the HS cohort was deletion of CFA 16, with a 6 Mb segment of this chromosome (extending from 47-53 Mb) deleted in 86% of all cases (80.4% of BMD and 96.7% of FCR). These data were supported by FISH analysis (see Figure 3 for an example) and LOH analysis on a subset of 26 BMDs and 20 FCRs for which matched blood and tumor DNA samples were available (data not shown). The second most frequently observed CNA in our cohort was deletion of CFA 22q11 at ~60.5 Mb, which includes the tumor suppressor gene *RB1*. DNA copy number loss of this region was identified in 55.8% of all cases, with deletions twice as common in the FCR (83.3% of FCRs and 41.1% of BMDs). The *TP53 *tumor suppressor gene locus (CFA 5q21 at ~35.5 Mb) was gained in 26.7% of all cases, with the frequency in the FCR (40%) being twice that of the BMD (19.6%). Further, gain of this region was almost three times more frequent than loss (26.7% gain vs 9.3% loss) across all cases and also within both breeds when considered separately (10.7% loss in BMDs, 6.6% loss in FCRs). aCGH indicated that deletion of the full length of CFA 26 was evident in approximately 10-20% of cases. In both breeds, however, deletion of the distal end of CFA 26, a region that contains *PTEN*, was deleted in 40.7% of cases (42.9% of BMD, 36.7% of FCR), representing the highest frequency of loss along this chromosome. FISH analysis with BAC clones representing *CDNK2A/B*, *RB1*, *TP53 *and *PTEN *supported the copy number status identified by aCGH in those cases evaluated.

## Discussion

We hypothesized that spontaneous canine HS exhibit recurrent CNAs of genes involved in histiocytic cancerization, and that identification of these CNAs may advance our understanding of the molecular characteristics of these cancers in both canine and human patients. The pathophysiologies of several dog and human cancers share many similarities and our previous studies have demonstrated that CNAs in a variety of human cancers are evolutionarily conserved in the corresponding canine cancer [40-42]. These findings support the idea of a fundamental and evolutionarily conserved association between cytogenetic abnormalities and tumor phenotype, indicating similar biological consequences in both species [41]. In testing our hypothesis we recruited client owned BMD and FCR patients, each with a confirmed diagnosis of HS, and performed genome-integrated aCGH analysis of 104 cases to identify recurrent CNAs at 1 Mb resolution. We also performed statistical analysis of clinical and demographic data from our HS cohort in order to expand knowledge of the epidemiological basis of this disease in these breeds.

Disseminated canine HS was first described as malignant histiocytosis in the BMD [19,20], and while this condition has been documented in other breeds [17], it appears that this clinical form continues to be reported more frequently in the BMD than other breeds. Conversely, Fidel et al. (2006) [25] described that the majority of HS in the FCR were restricted to a joint and/or muscle/skin, corresponding to a localized form of HS. Epidemiological data from our cohort are consistent with previous reports. Our data showed also that the anatomical location of the histiocytic tumors differed significantly between the two breeds investigated: BMDs present more frequently with tumors of internal organs and also with a high frequency of dissemination, while FCRs more often develop a localized tumor of the skin or leg. To our knowledge this study represents the first to provide statistical significance for these parameters. The contrasting patterns of anatomical location of HS in the BMD and FCR suggest that the genetic backgrounds of these two breeds may play a key role in determining risk, location and progression of this neoplasm, which could be assessed using genome wide association analyses. Prior human and mouse studies have identified specialized DC subtypes with heterogeneous functions [43] and so it is possible that the HS diagnosed in FCR and BMD represent malignancies of different DC subtypes, which might explain the different behavior of these cancers in the two breeds.

### Identification of breed-associated genomic copy number aberrations

No statistical differences were found between CNAs detected in HS of BMDs from two distinct geographic areas (France/USA). These data suggest that it is reasonable to sample BMDs from different geographic areas to increase the number of cases available for subsequent statistical analyses. This is not surprising considering the BMD has its roots in Switzerland in the late 19^th ^century, was admitted to the AKC registry in 1937, and experienced numerous international 'line-exchanges' over the ensuing 74 years. These data suggest a relatively homogeneous international population, supporting the conclusion of Quignon et al. [44] who proposed the use of international BMD cohorts for genetic studies. From a population genetics perspective, since the main populations of BMD are located in the USA and Europe, and there are no apparent differences in CNAs evident at 1 Mb resolution between the two BMD populations, we surmise that the genomic changes associated with HS are common to all BMDs regardless of geographic origin. This in turn may indicate that any risk factors for the development and progression of HS are linked tightly to the genetic makeup of the breed and independent of geography. Advances in understanding of the biological mechanism of HS in BMDs in the USA should therefore apply also to BMDs in other countries, extending the value of such studies.

Most of the CNAs identified in this study were common to both the BMD and the FCR, and so it is likely that such aberrations may also be evident in other breeds presenting with these malignancies. We identified 13 regions of the genome on seven chromosomes that showed a significant association between DNA copy number and breed of the patient (Table 1). Further, PCA indicated a significant association between specific CNAs and either breed (BMD/FCR) or anatomical location(s) of the tumor(s). Since breed and tumor location are so closely correlated, it is not possible from this study to determine whether the association between CNAs and breed was driven by breed itself or by the anatomical location of the tumor in that breed. Aberrations may be associated with location of affected organ/tissue, and/or dissemination/metastatic nature of HS. These aberrations could confer a proliferative advantage to the tumor in one particular organ, or elevate the risk of metastasis. An alternative hypothesis is that some of these aberrations are linked specifically with the genetic background of each breed. Since it has been shown previously that individual genetic backgrounds, as defined by breed in dogs, influence tumor karyotypes [40,45], we could hypothesize that some pathways are inactivated by germline mutations in one breed, creating a genetic risk for HS, but are inactivated by somatic modifications (genomic loss, mutation) in the second breed. Evaluation of HS in additional breeds that also present with a disseminated form of the disease, such as the Rottweiler, will aid in determining whether the apparent separation between BMD and FCR is driven by breed or by anatomical features of the cancer [9].

### Identification of highly recurrent CNAs shared by the FCR and the BMD - candidate regions for human HS

Despite the high level of genome reorganization evident in canine HS and the varying anatomical location of the tumors between breeds, we identified numerous CNAs within our sample population shared between the both breeds. Several of these were classified as recurrent (≥30% frequency) or highly recurrent (≥50% frequency). Among the most highly recurrent CNAs detected were loss of regions of CFA 2, CFA 11, CFA 16, CFA 22 and CFA 31, all of which were highly frequent (50-86%) in both breeds. The presence of highly recurrent abnormalities common to both breeds, along with their presence in both the localized and disseminated forms of HS, is suggestive of an association more with the cancer phenotype than with breed. These regions likely contain genes that may play a key role in malignant transformation of histiocytes, independent of anatomical location. This is especially so for the most frequent CNA, deletion of CFA 16, an aberration that was detected in 86% of HS cases (80.4% of BMD, 96.7% of FCR).

At first glance many of the recurrent aberrations identified in this study involved large contiguous tracts of the canine genome (Figures 4 and 5), and so identification of candidate genes is challenging. Closer consideration of subchromosomal differences in aberration frequency may however be used to determine minimal regions of interest. For example, Figures 4 and 5 indicate that while the full length of CFA 16 is deleted in at least 50% of all HS cases (both FCR and BMD), there are regional differences in the frequency of deletion along the length of the chromosome. The highest frequency of recurrent deletion (86%) along CFA 16 involved a 6 Mb region (47-53 Mb) towards the telomeric end of the chromosome. There are several annotated candidate genes within this region of the canine genome http://genome.ucsc.edu/cgi-bin/hgGateway?db=canFam2 that are known either to be involved in regulation of apoptosis, or which are suspected to be tumor suppressor genes; including *CDKN2A interacting protein (CDKN2AIP)*, *FAT tumor suppressor homolog1 *(*FAT1*), *Tumor suppressor candidate 3 *(*TUSC3*), *Mitochondrial Tumor Suppressor gene 1 *(*MTUS1*) and *pericentriolar material-1 *(*PCM1*). Of comparative significance specific to HS, *CDKN2AIP *is known to interact with *CDKN2A/p14ARF, TP53*/p53 and *MDM2*, all of which have been shown to be involved in human histiocytic disorders [4,8,9,11-14,46] and so this merits further investigation in future studies.

Similarly, a neighboring 2.4 Mb region of CFA 16 (41.8 Mb-44.2 Mb) was deleted in 84.9% of HS cases. Of possible comparative significance, this region of CFA 16 is in part orthologous to human chromosome (HSA) 8p22-p21.3, a region that is frequently deleted in numerous tumors, including multiple myeloma, prostate cancer, hepatocellular carcinoma and neck squamous cell carcinoma [47-50]. These data indicate that in both human and canine cancers, this region exhibits a strikingly high and comparable level of CNA. Further evaluation of both human and canine patients will be required to determine whether this shared deletion contains genes and regulatory elements associated with HS, or if its presence is merely a generalized passenger aberration.

### aCGH profiling of canine HS suggests that disruption of the p53 and Rb pathways is a common event

Segments of CFA 11 (q22), 22 (q11) and 26 (q25) all showed a high incidence of copy number loss in canine HS. Each of these three regions contain key cancer associated genes involved in the p53 and Rb pathways: *CDKN2A/B *(CFA 11q22), *RB1 *(CFA 22q11) and *PTEN *(CFA 26q25). *CDKN2 *encodes three distinct tumor suppressor genes (*ARF, p15*^*INK4b *^*p16*^*INK4a*^) that code for proteins regulating cell cycle progression via the Rb and p53 pathways. While p15^INK4b ^and p16^INK4a ^regulate the Rb-pathway, ARF inactivates MDM2 protein and so regulates p53 [51,52]. The human region orthologous to CFA 11q22 is HSA 9p21, which is among the most frequent sites of DNA copy number loss in human cancers [53]. Genes within this region, especially *p16*^*INK4a*^, have also been shown to be inactivated in several dog cancers including lymphoma, melanoma, hemangiosarcoma and osteosarcoma [42,54-59]. Since direct inactivation of *p16*^*INK4a *^by point mutation, deletion or promoter methylation is evident in approximately one third of human hematopoietic tumors [53,60], it is not surprising to find this locus is involved in human histiocytic disorders. Significantly, monosomy of HSA 9, including the *CDKN2 *locus, has been observed in different human dendritic proliferations including plasmacytoid DC sarcoma [8] and follicular DC sarcoma [9]. Moreover deletion of HSA 9p has been reported as the second most frequently observed aberration in Langerhans cell histiocytosis (LCH) of the lung [10]. In mice, loss of *INK4a *allows macrophages to bypass senescence [61] and Pten and Ink4a/Arf have a cooperative role in restricting macrophage growth. The same is true in human HS where inactivation of *PTEN *and *INK4a/ARF *tumor suppressors are critical steps in the pathogenesis of this cancer [4,11]. It is therefore of interest that in this study >50% of HS cases presented with a deletion of the *CDKN2 *locus. Other mechanisms, such as DNA sequence mutations or methylation, may also inactivate these tumor suppressor genes. In future studies it will be important to investigate whether HS cases presenting with no apparent deletion of *CDKN2 *have an increased rate of DNA sequence mutation of this locus, resulting in aberrant expression for reasons other than gene dosage.

Also belonging to these key pathways is the gene *Retinoblastoma 1 *(*RB1*), which is disrupted in a variety of human solid tumors including pituitary adenomas, esophageal carcinoma, gliomas and ovarian cancer [62]. Deletion of HSA 13q14, containing *RB1*, is also a common event in a wide variety of acute/chronic myeloid disorders as well as in human dendritic sarcomas (plasmacytoid DC and follicular DC sarcomas) [8,63]. In our study, loss of the *RB1 *locus on CFA 22 was highly recurrent across the cohort (55.8% of HS cases) although it was detected twice as frequently in HS tumors of FCRs than of BMDs (83.3% of FCR cases vs 41.1% of BMD cases).

Deletion of HSA 17p, containing *TP53*, has been described in human LCH [10,12], while other studies reported an elevated expression of p53 in this disease [13,14]. In the present study, 36% of canine HS cases demonstrated CNA of *TP53*, representing copy number loss in 9.3% cases and gain in 26.7% of cases. Further studies are needed to assess if gene dosage of *TP53*, as well as other mechanisms, result in altered expression of p53 that may be correlated with elevated expression of this protein in Langerhans cell proliferation.

Deletion of CFA26 was present in approximately 20% of canine HS cases, but the telomeric end of this chromosome, a region encoding the *PTEN *locus, was deleted in ~41% of cases, regardless of breed. *PTEN *plays a significant role in inducing cycle arrest and programming apoptosis. It is an antagonist of the *PI3K/AKT *pathway, and in turn regulates the *Rb *pathway [64]. It also controls p53 protein levels and transcriptional activity through both phosphatase-dependent and independent mechanisms [65]. *PTEN *has been shown to be deleted or mutated in a wide range of human tumors [64] and also in several canine tumors [58,66]. While *PTEN *influences p53 transcriptional activity and p53 stability [65], no association was found in our data between gain/loss of *TP53 *and *PTEN *loss (Fisher's Exact test, data not shown).

Copy number aberrations common to both breeds, combined with their likely consequential impact on the same pathways in human and canine HS, support the relevance of the dog as a model of HS. In addition to the genes discussed above we suspect that other tumor suppressors on CFA 2, CFA 16 and CFA 31 (deleted in 50%, 86% and 61.6% of HS tumors, respectively) also may play an important role in histiocytic cancerization. Their identity likely will become apparent with increased resolution and functional analysis of genes within these regions.

## Conclusions

This study demonstrates that histiocytic sarcomas of two dog breeds with distinct genetic backgrounds (BMD and FCR), but sharing a high incidence of the disease, present with highly aberrant genome-wide DNA copy number profiles. The presence of numerous highly recurrent CNAs shared by both BMD and FCR tumors suggests that these are associated more with the cancer phenotype than breed. The small number of breed associated CNAs identified may contribute to the major differences in the varying tumor location evident in these two breeds. The most highly recurrent aberrations revealed in this study are evolutionarily conserved with those reported in human histiocytic proliferations, suggesting that human and dog HS share a conserved pathogenesis. The breed associated clinical features and chromosomal aberrations of canine HS offer a valuable spontaneous model for the human counterpart, aiding elucidation of the pathophysiological and genetic mechanisms associated with histiocytic malignancies and providing new opportunities for developing effective therapeutic modalities for both species.

## Competing interests

The authors declare that they have no competing interests.

## Authors' contributions

MB conceptualized and designed the project. MB and CA obtained all the samples for the study, for which JC and JA conducted the pathology review. BH performed and analyzed the aCGH, FISH and LOH data with input from RT and MB. AMR was responsible for statistical design. BH and AMR conducted the statistical evaluation of the aCGH data. BH, MB and RT wrote the manuscript. All authors read, edited and approved the final manuscript.

## Pre-publication history

The pre-publication history for this paper can be accessed here:

http://www.biomedcentral.com/1471-2407/11/201/prepub

## Supplementary Material

Additional file 1 **Table S1. List of microsatellite markers used for LOH study**. The position of each sequence is shown as the base pair location in the canine genome assembly, canFam2. Forward primers of CFA 11 markers had an M13-tail and were used with an M13 primer fluorescently tagged at the 5' end either with PET, VIC, FAM or NED to facilitate multiplexing. Click here for file

Additional file 2 **Table S2. Signalment and clinical data for all 125 canine histiocytic sarcoma cases for which unfixed tumor biopsies were available**. Unshaded cases indicate 86 cases used for aCGH data analysis. Cases that did not yield sufficient quality DNA and those that did not contain evident CNAs are highlighted in grey. Click here for file

## References

[B1] Jaffe ES Pathology and genetics of tumours of haematopeietic tissues 2001 Lyon: World Health Organization of Tumours. International Agency for Research on Cancer

[B2] Pileri SA Grogan TM Harris NL Banks P Campo E Chan JK Favera RD Delsol G De Wolf-Peeters C Falini B Tumours of histiocytes and accessory dendritic cells: an immunohistochemical approach to classification from the International Lymphoma Study Group based on 61 cases Histopathology 2002 41 1 1 29 10.1046/j.1365-2559.2002.01418.x 12121233

[B3] Favara BE Feller AC Pauli M Jaffe ES Weiss LM Arico M Bucsky P Egeler RM Elinder G Gadner H Contemporary classification of histiocytic disorders. The WHO Committee On Histiocytic/Reticulum Cell Proliferations. Reclassification Working Group of the Histiocyte Society Med Pediatr Oncol 1997 29 3 157 166 10.1002/(SICI)1096-911X(199709)29:3<157::AID-MPO1>3.0.CO;2-C 9212839

[B4] Carrasco DR Fenton T Sukhdeo K Protopopova M Enos M You MJ Di Vizio D Nogueira C Stommel J Pinkus GS The PTEN and INK4A/ARF tumor suppressors maintain myelolymphoid homeostasis and cooperate to constrain histiocytic sarcoma development in humans Cancer Cell 2006 9 5 379 390 10.1016/j.ccr.2006.03.028 16697958

[B5] Teyssier JR Behar C Pignon B Caulet T Patey M Bajolle F Adnet JJ Chromosomal changes in a documented case of malignant histiocytosis: significance of polyploidy Cancer Genet Cytogenet 1986 21 1 85 91 10.1016/0165-4608(86)90203-7 3510716

[B6] Mecucci C Donti E Tabilio A Martelli MF Van den Berghe H Clinical and cytogenetic findings in monocyte--macrophage system malignancies with initial spontaneous regression Cancer Genet Cytogenet 1983 9 4 317 327 10.1016/0165-4608(83)90079-1 6871836

[B7] Schouten TJ Hustinx TW Scheres JM Holland R de Vaan GA Malignant histiocytosis. Clinical and cytogenetic studies in a newborn and a child Cancer 1983 52 7 1229 1236 10.1002/1097-0142(19831001)52:7<1229::AID-CNCR2820520717>3.0.CO;2-N 6883287

[B8] Leroux D Mugneret F Callanan M Radford-Weiss I Dastugue N Feuillard J Le Mee F Plessis G Talmant P Gachard N CD4(+), CD56(+) DC2 acute leukemia is characterized by recurrent clonal chromosomal changes affecting 6 major targets: a study of 21 cases by the Groupe Francais de Cytogenetique Hematologique Blood 2002 99 11 4154 4159 10.1182/blood.V99.11.4154 12010820

[B9] Sander B Middel P Gunawan B Schulten HJ Baum F Golas MM Schulze F Grabbe E Parwaresch R Fuzesi L Follicular dendritic cell sarcoma of the spleen Hum Pathol 2007 38 4 668 672 10.1016/j.humpath.2006.08.030 17367608

[B10] Dacic S Trusky C Bakker A Finkelstein SD Yousem SA Genotypic analysis of pulmonary Langerhans cell histiocytosis Hum Pathol 2003 34 12 1345 1349 10.1016/j.humpath.2003.07.014 14691922

[B11] Kumar R Khan SP Joshi DD Shaw GR Ketterling RP Feldman AL Pediatric histiocytic sarcoma clonally related to precursor B-cell acute lymphoblastic leukemia with homozygous deletion of CDKN2A encoding p16(INK4A) Pediatr Blood Cancer 2010 10.1002/pbc.22810 20973102

[B12] Murakami I Gogusev J Fournet JC Glorion C Jaubert F Detection of molecular cytogenetic aberrations in langerhans cell histiocytosis of bone Hum Pathol 2002 33 5 555 560 10.1053/hupa.2002.124035 12094383

[B13] Bank MI Rengtved P Carstensen H Petersen BL p53 expression in biopsies from children with Langerhans cell histiocytosis J Pediatr Hematol Oncol 2002 24 9 733 736 10.1097/00043426-200212000-00010 12468914

[B14] Weintraub M Bhatia KG Chandra RS Magrath IT Ladisch S p53 expression in Langerhans cell histiocytosis J Pediatr Hematol Oncol 1998 20 1 12 17 10.1097/00043426-199801000-00002 9482407

[B15] De Pas T Spitaleri G Pruneri G Curigliano G Noberasco C Luini A Andreoni B Testori A de Braud F Dendritic cell sarcoma: an analytic overview of the literature and presentation of original five cases Crit Rev Oncol Hematol 2008 65 1 1 7 10.1016/j.critrevonc.2007.06.003 17658269

[B16] Mitelman F Johansson B Mertens F The impact of translocations and gene fusions on cancer causation Nat Rev Cancer 2007 7 4 233 245 10.1038/nrc2091 17361217

[B17] Affolter VK Moore PF Localized and disseminated histiocytic sarcoma of dendritic cell origin in dogs Vet Pathol 2002 39 1 74 83 10.1354/vp.39-1-74 12102221

[B18] Moore PF Affolter VK Vernau W Canine hemophagocytic histiocytic sarcoma: a proliferative disorder of CD11d+ macrophages Vet Pathol 2006 43 5 632 645 10.1354/vp.43-5-632 16966440

[B19] Moore PF Rosin A Malignant histiocytosis of Bernese mountain dogs Vet Pathol 1986 23 1 1 10 3946051 10.1177/030098588602300101

[B20] Rosin A Moore P Dubielzig R Malignant histiocytosis in Bernese Mountain dogs J Am Vet Med Assoc 1986 188 9 1041 1045 3710888

[B21] Dobson J Villiers E Roulois A Gould S Mellor P Hoather T Watson P Histiocytic sarcoma of the spleen in flat-coated retrievers with regenerative anaemia and hypoproteinaemia Vet Rec 2006 158 24 825 829 10.1136/vr.158.24.825 16782856

[B22] Padgett GA Madewell BR Keller ET Jodar L Packard M Inheritance of histiocytosis in Bernese mountain dogs J Small Anim Pract 1995 36 3 93 98 10.1111/j.1748-5827.1995.tb02838.x 7783441

[B23] Abadie J Hedan B Cadieu E De Brito C Devauchelle P Bourgain C Parker HG Vaysse A Margaritte-Jeannin P Galibert F Epidemiology, Pathology, and Genetics of Histiocytic Sarcoma in the Bernese Mountain Dog Breed J Hered 2009 10.1093/jhered/esp039 PMC3139364 19531730

[B24] Dobson J Hoather T McKinley TJ Wood JL Mortality in a cohort of flat-coated retrievers in the UK Vet Comp Oncol 2009 7 2 115 121 10.1111/j.1476-5829.2009.00181.x 19453365

[B25] Fidel J Schiller I Hauser B Jausi Y Rohrer-Bley C Roos M Kaser-Hotz B Histiocytic sarcomas in flat-coated retrievers: a summary of 37 cases (November 1998-March 2005) Vet Comp Oncol 2006 4 2 63 74 10.1111/j.1476-5810.2006.00090.x 19754816

[B26] Thomas R Duke SE Karlsson EK Evans A Ellis P Lindblad-Toh K Langford CF Breen M A genome assembly-integrated dog 1 Mb BAC microarray: a cytogenetic resource for canine cancer studies and comparative genomic analysis Cytogenet Genome Res 2008 122 2 110 121 10.1159/000163088 19096206 PMC2874680

[B27] Lindblad-Toh K Wade CM Mikkelsen TS Karlsson EK Jaffe DB Kamal M Clamp M Chang JL Kulbokas EJ Zody MC Genome sequence, comparative analysis and haplotype structure of the domestic dog Nature 2005 438 7069 803 819 10.1038/nature04338 16341006

[B28] Thomas R Duke SE Bloom SK Breen TE Young AC Feiste E Seiser EL Tsai PC Langford CF Ellis P A cytogenetically characterized, genome-anchored 10-Mb BAC set and CGH array for the domestic dog J Hered 2007 98 5 474 484 10.1093/jhered/esm053 17702974

[B29] Jong K Marchiori E Meijer G Vaart AV Ylstra B Breakpoint identification and smoothing of array comparative genomic hybridization data Bioinformatics 2004 20 18 3636 3637 10.1093/bioinformatics/bth355 15201182

[B30] Boutin-Ganache I Raposo M Raymond M Deschepper CF M13-tailed primers improve the readability and usability of microsatellite analyses performed with two different allele-sizing methods Biotechniques 2001 31 1 24 26 28 11464515

[B31] Clark LA Tsai KL Steiner JM Williams DA Guerra T Ostrander EA Galibert F Murphy KE Chromosome-specific microsatellite multiplex sets for linkage studies in the domestic dog Genomics 2004 84 3 550 554 10.1016/j.ygeno.2004.06.006 15498461

[B32] Breen M Bullerdiek J Langford CF The DAPI banded karyotype of the domestic dog (Canis familiaris) generated using chromosome-specific paint probes Chromosome Research 1999 7 5 401 406 10.1023/A:1009224232134 10515215

[B33] Breen M Hitte C Lorentzen TD Thomas R Cadieu E Sabacan L Scott A Evanno G Parker HG Kirkness EF An integrated 4249 marker FISH/RH map of the canine genome BMC Genomics 2004 5 1 65 10.1186/1471-2164-5-65 15363096 PMC520820

[B34] Reich D Price AL Patterson N Principal component analysis of genetic data Nat Genet 2008 40 5 491 492 10.1038/ng0508-491 18443580

[B35] Price AL Zaitlen NA Reich D Patterson N New approaches to population stratification in genome-wide association studies Nat Rev Genet 2010 11 7 459 463 20548291 10.1038/nrg2813 PMC2975875

[B36] Reich DE Goldstein DB Detecting association in a case-control study while correcting for population stratification Genet Epidemiol 2001 20 1 4 16 10.1002/1098-2272(200101)20:1<4::AID-GEPI2>3.0.CO;2-T 11119293

[B37] Hoggart CJ Parra EJ Shriver MD Bonilla C Kittles RA Clayton DG McKeigue PM Control of confounding of genetic associations in stratified populations Am J Hum Genet 2003 72 6 1492 1504 10.1086/375613 12817591 PMC1180309

[B38] Pritchard JK Stephens M Rosenberg NA Donnelly P Association mapping in structured populations Am J Hum Genet 2000 67 1 170 181 10.1086/302959 10827107 PMC1287075

[B39] Dwass M Modified Randomization Tests for Nonparametric Hypotheses The Annals of Mathematical Statistics 1957 28 181 187 10.1214/aoms/1177707045

[B40] Thomas R Duke SE Wang HJ Breen TE Higgins RJ Linder KE Ellis P Langford CF Dickinson PJ Olby NJ 'Putting our heads together': insights into genomic conservation between human and canine intracranial tumors J Neurooncol 2009 94 3 333 349 10.1007/s11060-009-9877-5 19333554 PMC3225023

[B41] Breen M Modiano JF Evolutionarily conserved cytogenetic changes in hematological malignancies of dogs and humans--man and his best friend share more than companionship Chromosome Res 2008 16 1 145 154 10.1007/s10577-007-1212-4 18293109

[B42] Fosmire SP Thomas R Jubala CM Wojcieszyn JW Valli VE Getzy DM Smith TL Gardner LA Ritt MG Bell JS Inactivation of the p16 cyclin-dependent kinase inhibitor in high-grade canine non-Hodgkin's T-cell lymphoma Vet Pathol 2007 44 4 467 478 10.1354/vp.44-4-467 17606508

[B43] Shortman K Liu YJ Mouse and human dendritic cell subtypes Nat Rev Immunol 2002 2 3 151 161 10.1038/nri746 11913066

[B44] Quignon P Herbin L Cadieu E Kirkness EF Hedan B Mosher DS Galibert F Andre C Ostrander EA Hitte C Canine population structure: assessment and impact of intra-breed stratification on SNP-based association studies PLoS ONE 2007 2 12 e1324 10.1371/journal.pone.0001324 18091995 PMC2129117

[B45] Tamburini BA Trapp S Phang TL Schappa JT Hunter LE Modiano JF Gene expression profiles of sporadic canine hemangiosarcoma are uniquely associated with breed PLoS ONE 2009 4 5 e5549 10.1371/journal.pone.0005549 19461996 PMC2680013

[B46] Dakic A Wu L Hemopoietic precursors and development of dendritic cell populations Leuk Lymphoma 2003 44 9 1469 1475 10.1080/1042819031000083370 14565646

[B47] Sutlu T Alici E Jansson M Wallblom A Dilber MS Gahrton G Nahi H The prognostic significance of 8p21 deletion in multiple myeloma Br J Haematol 2009 144 2 266 268 10.1111/j.1365-2141.2008.07454.x 19016723

[B48] Hornstein M Hoffmann MJ Alexa A Yamanaka M Muller M Jung V Rahnenfuhrer J Schulz WA Protein phosphatase and TRAIL receptor genes as new candidate tumor genes on chromosome 8p in prostate cancer Cancer Genomics Proteomics 2008 5 2 123 136 18460741

[B49] Di Benedetto M Pineau P Nouet S Berhouet S Seitz I Louis S Dejean A Couraud PO Strosberg AD Stoppa-Lyonnet D Mutation analysis of the 8p22 candidate tumor suppressor gene ATIP/MTUS1 in hepatocellular carcinoma Mol Cell Endocrinol 2006 252 1-2 207 215 10.1016/j.mce.2006.03.014 16650523

[B50] Ye H Pungpravat N Huang BL Muzio LL Mariggio MA Chen Z Wong DT Zhou X Genomic assessments of the frequent loss of heterozygosity region on 8p21.3-p22 in head and neck squamous cell carcinoma Cancer Genet Cytogenet 2007 176 2 100 106 10.1016/j.cancergencyto.2007.04.003 17656251 PMC2000851

[B51] Kim WY Sharpless NE The regulation of INK4/ARF in cancer and aging Cell 2006 127 2 265 275 10.1016/j.cell.2006.10.003 17055429

[B52] Gil J Peters G Regulation of the INK4b-ARF-INK4a tumour suppressor locus: all for one or one for all Nat Rev Mol Cell Biol 2006 7 9 667 677 16921403 10.1038/nrm1987

[B53] Sharpless NE INK4a/ARF: a multifunctional tumor suppressor locus Mutat Res 2005 576 1-2 22 38 15878778 10.1016/j.mrfmmm.2004.08.021

[B54] Koenig A Bianco SR Fosmire S Wojcieszyn J Modiano JF Expression and significance of p53, rb, p21/waf-1, p16/ink-4a, and PTEN tumor suppressors in canine melanoma Vet Pathol 2002 39 4 458 472 10.1354/vp.39-4-458 12126149

[B55] Levine RA Fleischli MA Inactivation of p53 and retinoblastoma family pathways in canine osteosarcoma cell lines Vet Pathol 2000 37 1 54 61 10.1354/vp.37-1-54 10643981

[B56] Modiano JF Breen M Valli VE Wojcieszyn JW Cutter GR Predictive value of p16 or Rb inactivation in a model of naturally occurring canine non-Hodgkin's lymphoma Leukemia 2007 21 1 184 187 10.1038/sj.leu.2404392 16990767

[B57] Yonemaru K Sakai H Murakami M Kodama A Mori T Yanai T Maruo K Masegi T The significance of p53 and retinoblastoma pathways in canine hemangiosarcoma J Vet Med Sci 2007 69 3 271 278 10.1292/jvms.69.271 17409643

[B58] Dickerson EB Thomas R Fosmire SP Lamerato-Kozicki AR Bianco SR Wojcieszyn JW Breen M Helfand SC Modiano JF Mutations of phosphatase and tensin homolog deleted from chromosome 10 in canine hemangiosarcoma Vet Pathol 2005 42 5 618 632 10.1354/vp.42-5-618 16145208

[B59] Thomas R Scott A Langford CF Fosmire SP Jubala CM Lorentzen TD Hitte C Karlsson EK Kirkness E Ostrander EA Construction of a 2-Mb resolution BAC microarray for CGH analysis of canine tumors Genome Res 2005 15 12 1831 1837 10.1101/gr.3825705 16339382 PMC1356122

[B60] Ruas M Peters G The p16INK4a/CDKN2A tumor suppressor and its relatives Biochim Biophys Acta 1998 1378 2 F115 177 9823374 10.1016/s0304-419x(98)00017-1

[B61] Randle DH Zindy F Sherr CJ Roussel MF Differential effects of p19(Arf) and p16(Ink4a) loss on senescence of murine bone marrow-derived preB cells and macrophages Proc Natl Acad Sci USA 2001 98 17 9654 9659 10.1073/pnas.171217498 11481442 PMC55507

[B62] Presneau N Manderson EN Tonin PN The quest for a tumor suppressor gene phenotype Curr Mol Med 2003 3 7 605 629 10.2174/1566524033479500 14601636

[B63] Della Porta M Rigolin GM Bugli AM Bardi A Bragotti LZ Bigoni R Cuneo A Castoldi G Differentiation of follicular dendritic sarcoma cells into functional myeloid-dendritic cell-like elements Eur J Haematol 2003 70 5 315 318 10.1034/j.1600-0609.2003.00042.x 12694168

[B64] Di Cristofano A Pandolfi PP The multiple roles of PTEN in tumor suppression Cell 2000 100 4 387 390 10.1016/S0092-8674(00)80674-1 10693755

[B65] Freeman DJ Li AG Wei G Li HH Kertesz N Lesche R Whale AD Martinez-Diaz H Rozengurt N Cardiff RD PTEN tumor suppressor regulates p53 protein levels and activity through phosphatase-dependent and -independent mechanisms Cancer Cell 2003 3 2 117 130 10.1016/S1535-6108(03)00021-7 12620407

[B66] Thomas R Wang HJ Tsai PC Langford CF Fosmire SP Jubala CM Getzy DM Cutter GR Modiano JF Breen M Influence of genetic background on tumor karyotypes: evidence for breed-associated cytogenetic aberrations in canine appendicular osteosarcoma Chromosome Res 2009 17 3 365 377 10.1007/s10577-009-9028-z 19337847 PMC3758998

