# Supplementary material for: Molecular cytogenetic characterization of canine histiocytic sarcoma: A spontaneous model for human histiocytic cancer identifies deletion of tumor suppressor genes and highlights influence of genetic background on tumor behavior
Source: BMC Cancer. 2011 May 26;11:201. doi: 10.1186/1471-2407-11-201 (PMC3121728; doi:10.1186/1471-2407-11-201)
Supplement: Additional file 1 — Table S1. List of microsatellite markers used for LOH study. The position of each sequence is shown as the base pair location in the canine genome assembly, canFam2. Forward primers of CFA 11 markers had an M13-tail and were used with an M13 primer fluorescently tagged at the 5' end either with PET, VIC, FAM or NED to facilitate multiplexing. [file 1471-2407-11-201-S1.DOC]

Additional File 1: Table S1: List of microsatellite markers used for LOH study. The position of each sequence is shown as the base pair location in the canine genome assembly, CanFam2. Forward primers of CFA11 markers had an M13-tail and were used with an M13 primer fluorescently tagged at the 5’ end either with PET, VIC, FAM or NED to facilitate multiplexing.

| CFA | Marker | Position | Size  of product | Forward primer | Reverse primer |
| --- | --- | --- | --- | --- | --- |
| CFA 16 | REN85N14 | chr16:22227366-22227600 | 235 | AAGGCAGGAGGAGGAGCAC | TATGGAGATGGAGGGCACAC |
|  | FH2175 | chr16:34805975-34806227 | 253 | TTCATTGATTTCTCCATTGGC | AGGACTCTAAAAACTTGCCTCC |
|  | REN275L19 | chr16:41816906-41817133 | 228 | CCTGCATAATCCTGCAATGA | CTATCCTGCTGCACCTAGCC |
|  | FH2155 | chr16:54088246-54088744 | 499 | TGTAGATGATGGAGACATTGGG | AGGCAAATATGCCAAGGATG |
|  | REN214L11 | chr16:5800087-5800316 | 230 | AAATTGCAAATCTTGGCCC | TCCATGCTAAGACCCCCATA |
|  | FH3592 | chr16:60183232-60183656 | 425 | AGGTGCTGAGCATGTTATCC | GGGTCAAAGTGTTGACATGG |
| CFA 11 | FH2982 | chr11:41069406-41069780 | 375 | cacgacgttgtaaaacgacCATGACATTCTTGCTTTAGCC | GGGCAAAGGGTTTTCTAAGG |
|  | FH2706 | chr11:43991845-43992046 | 202 | cacgacgttgtaaaacgacACCTGAGCCAGAAATGAATG | AGATCTGCAGCATAGATGTGG |
|  | CFA11-45,5 | chr11:45530117-45530354 | 238 | cacgacgttgtaaaacgacCCACCCAACTTATCCTTCCA | CTCAAAGGGGCAGACAGAAG |
|  | CFA11-45,8 | chr11:45855105-45855503 | 399 | cacgacgttgtaaaacgacTGGGGTCCTTTCACTCTATGA | GAAGCAGCAGTGTACTGATTGAA |
|  | CFA11-48,6 | chr11:48631831-48632065 | 235 | cacgacgttgtaaaacgacGGGACTCAAAGGTTGCACAT | AACGGGGGTATAGCTGTGTG |
|  | CFA11-49,1 | chr11:49165387-49165619 | 233 | cacgacgttgtaaaacgacAGTCCTGAATTTGCCAAAGG | CCCCCTTAGCAATAGCCTTG |
|  | CFA11-49,7 | chr11:49792263-49792507 | 245 | cacgacgttgtaaaacgacCACTAAATAAAATGGTAAATTCCACA | AAAACATAGTTACTTTGATCCTGAAGT |
