# Supplementary material for: Molecular cytogenetic characterization of canine histiocytic sarcoma: A spontaneous model for human histiocytic cancer identifies deletion of tumor suppressor genes and highlights influence of genetic background on tumor behavior
Source: BMC Cancer. 2011 May 26;11:201. doi: 10.1186/1471-2407-11-201 (PMC3121728; doi:10.1186/1471-2407-11-201)
Supplement: Additional file 2 — Table S2. Signalment and clinical data for all 125 canine histiocytic sarcoma cases for which unfixed tumor biopsies were available. Unshaded cases indicate 86 cases used for aCGH data analysis. Cases that did not yield sufficient quality DNA and those that did not contain evident CNAs are highlighted in grey. [file 1471-2407-11-201-S2.DOC]

Additional File 2: Table S2: Signalment and clinical data for all 125 canine histiocytic sarcoma cases for which tumor biopsies were available. Unshaded cases are those 86 which produced aCGH data used in the analysis. Cases that did not yield sufficient quality DNA and those that did not contain evident CNAs are highlighted in grey.

| case # | Breed | Gender | Age at diagnosis (years) | Location of tumor | origin |
| --- | --- | --- | --- | --- | --- |
| BMD01 | BMD | M | 7 | spleen, liver | USA |
| BMD02 | BMD | M | 6 | lung | USA |
| BMD03 | BMD | F | 9 | lung, spleen, femur, liver, lymph node | USA |
| BMD04 | BMD | F | 10 | NA | USA |
| BMD05 | BMD | F | 4 | thoracic mass | USA |
| BMD06 | BMD | M | 8 | spleen, lung | USA |
| BMD07 | BMD | M | 9 | abdominal mass | USA |
| BMD08 | BMD | F | 7 | lung | USA |
| BMD09 | BMD | F | 9 | lung, lymph node | USA |
| BMD10 | BMD | M | 7 | lung | USA |
| BMD11 | BMD | M | 8 | lymph node | USA |
| BMD12 | BMD | F | 8 | axillary mass | USA |
| BMD13 | BMD | M | 8 | liver | USA |
| BMD14 | BMD | F | 7 | liver, lung | USA |
| BMD15 | BMD | F | 7 | lung | USA |
| BMD16 | BMD | F | 11 | lung | USA |
| BMD17 | BMD | M | 8 | skin | USA |
| BMD18 | BMD | M | 7 | lung | USA |
| BMD19 | BMD | M | 5 | limb, lung | USA |
| BMD20 | BMD | F | 9 | spleen, liver | USA |
| BMD21 | BMD | M | 8 | spleen, liver, lung, lymph node | USA |
| BMD22 | BMD | M | 7 | lung | USA |
| BMD23 | BMD | M | 9 | lung | USA |
| BMD24 | BMD | M | 6 | skin, bone | USA |
| BMD25 | BMD | F | 10 | spleen | USA |
| BMD26 | BMD | M | 7 | liver | USA |
| BMD27 | BMD | M | 10 | spleen, liver, lymph node | USA |
| BMD28 | BMD | F | 6 | spleen | USA |
| BMD29 | BMD | M | NA | abdominal mas | USA |
| BMD30 | BMD | M | 9 | NA | USA |
| BMD31 | BMD | F | 8 | skin, lung | USA |
| BMD32 | BMD | M | 9 | lung, spleen, mediastinal lymph node | France |
| BMD33 | BMD | M | 5 | liver, lung | France |
| BMD34 | BMD | F | 9 | spleen, liver, lung, lymph node | France |
| BMD35 | BMD | M | 6 | spleen | France |
| BMD36 | BMD | F | 6 | mediastinal lymph node, liver | France |
| BMD37 | BMD | F | 5 | spleen, lymph node, lung | France |
| BMD38 | BMD | M | 5 | lung, liver, spleen, lymph node | France |
| BMD39 | BMD | M | 5 | lung, lymph node | France |
| BMD40 | BMD | F | 6 | spleen,liver, lung | France |
| BMD41 | BMD | M | 5 | lung, lymph node | France |
| BMD42 | BMD | M | 6 | liver, lung, lymph node | France |
| BMD43 | BMD | M | 5 | liver, spleen | France |
| BMD44 | BMD | F | 8 | lung | France |
| BMD45 | BMD | M | 7 | liver, lymph node | France |
| BMD46 | BMD | M | 7 | liver, spleen | France |
| BMD47 | BMD | F | 3 | cutaneous mass | France |
| BMD48 | BMD | M | 6 | lung, lymph node | France |
| BMD49 | BMD | M | 9 | limb | France |
| BMD50 | BMD | M | 9 | lung | France |
| BMD51 | BMD | M | 7 | lung | France |
| BMD52 | BMD | F | 8 | lymph node | France |
| BMD53 | BMD | F | 6 | liver | France |
| BMD54 | BMD | M | 6 | synovial tumor | France |
| BMD55 | BMD | M | 8 | lung | France |
| BMD56 | BMD | M | 8 | mediastinum, kidney | France |
| BMD57 | BMD | F | 6 | NA | USA |
| BMD58 | BMD | F | 9,5 | liver, lung | USA |
| BMD59 | BMD | F | 6 | skin, spleen | USA |
| BMD60 | BMD | M | NA | NA | USA |
| BMD61 | BMD | M | 8 | lung, liver | USA |
| BMD62 | BMD | F | 10,5 | lung, liver | USA |
| BMD63 | BMD | F | 10 | lung, spleen, liver | USA |
| BMD64 | BMD | F | 10 | lung | USA |
| BMD65 | BMD | F | 9,5 | liver, spleen | USA |
| BMD66 | BMD | F | 9,5 | lung | USA |
| BMD67 | BMD | M | 7 | liver, spleen | USA |
| BMD68 | BMD | M | 7 | spleen, lymph node | USA |
| BMD69 | BMD | M | 7 | liver, spleen | USA |
| BMD70 | BMD | F | 11 | lung | USA |
| BMD71 | BMD | F | 8 | lung | USA |
| BMD72 | BMD | F | 8 | lung, liver | USA |
| BMD73 | BMD | F | 10 | skin | USA |
| BMD74 | BMD | F | 2,5 | lung, liver | USA |
| BMD75 | BMD | M | 8,5 | lung, abdominal mass | USA |
| BMD76 | BMD | F | 8,5 | liver, spleen | USA |
| BMD77 | BMD | M | 7,5 | lung, spleen, liver | USA |
| BMD78 | BMD | M | 4 | spleen, lymph node | USA |
| BMD79 | BMD | M | 6 | spleen, liver | USA |
| BMD80 | BMD | F | 7,5 | spleen, liver | USA |
| BMD81 | BMD | M | 6,5 | spleen | USA |
| BMD82 | BMD | M | NA | lung, spleen | France |
| BMD83 | BMD | F | NA | lung, spleen | France |
| BMD84 | BMD | M | NA | liver | France |
| BMD85 | BMD | F | NA | lung | France |
| BMD86 | BMD | M | NA | spleen | France |
| BMD87 | BMD | F | NA | lung | France |
| BMD88 | BMD | M | NA | lymph node | France |
| BMD89 | BMD | M | NA | limb | France |
| FCR01 | FCR | F | 11 | spleen, liver, lung | USA |
| FCR02 | FCR | M | 6 | spleen, lymph node | USA |
| FCR03 | FCR | F | 7 | limb | USA |
| FCR04 | FCR | F | 8 | joint | USA |
| FCR05 | FCR | M | 9 | limb | USA |
| FCR06 | FCR | F | 11 | liver, lung | USA |
| FCR07 | FCR | M | 10 | liver | USA |
| FCR08 | FCR | M | 11 | axillary lymph node | USA |
| FCR09 | FCR | F | 11 | NA | USA |
| FCR10 | FCR | F | 7 | lung, kidney | USA |
| FCR11 | FCR | F | 5 | NA | USA |
| FCR12 | FCR | M | 6 | kidney | USA |
| FCR13 | FCR | F | 6 | lung | USA |
| FCR14 | FCR | F | 9 | limb | USA |
| FCR15 | FCR | F | 11 | limb | USA |
| FCR16 | FCR | M | 7 | limb | USA |
| FCR17 | FCR | M | 9 | limb | USA |
| FCR18 | FCR | F | 9 | limb | USA |
| FCR19 | FCR | M | 9 | spleen | USA |
| FCR20 | FCR | F | 8 | lung | USA |
| FCR21 | FCR | F | 9 | spleen, thoracic mass | USA |
| FCR22 | FCR | F | 10 | lung | USA |
| FCR23 | FCR | M | 10 | liver | USA |
| FCR24 | FCR | F | 9 | spleen, liver, kidney | USA |
| FCR25 | FCR | M | 5 | joint and lymph node | USA |
| FCR26 | FCR | M | 10 | limb | USA |
| FCR27 | FCR | F | 11 | axillary lymph node | USA |
| FCR28 | FCR | M | 8 | limb | USA |
| FCR29 | FCR | M | NA | spleen, kidney, lymph node | USA |
| FCR30 | FCR | F | 8 | axillary mass | USA |
| FCR31 | FCR | M | 11 | skin | USA |
| FCR32 | FCR | M | 5,5 | joint | USA |
| FCR33 | FCR | M | 6,5 | lymph node | USA |
| FCR34 | FCR | M | 9,5 | lung | USA |
| FCR35 | FCR | M | 9 | limb | USA |
| FCR36 | FCR | F | 7,5 | limb | USA |
